# Mapping the immunosuppressive environment in uterine tumors: implications for immunotherapy

**DOI:** 10.1007/s00262-014-1537-8

**Published:** 2014-03-22

**Authors:** Anke Vanderstraeten, Catherine Luyten, Godelieve Verbist, Sandra Tuyaerts, Frederic Amant

**Affiliations:** 1 Division of Gynecologic Oncology, Department of Oncology, Katholieke Universiteit Leuven, Herestraat 49, Box 611, 3000 Leuven, Belgium; 2 Division of Gynecologic Oncology, University Hospitals Leuven, Campus Gasthuisberg, Leuven, Belgium

**Keywords:** Immunosuppression, Uterine cancer, Immune checkpoints, IDO, Galectins, Myeloid-derived suppressor cells

## Abstract

The major hurdle for cancer vaccines to be effective is posed by tumor immune evasion. Several common immune mechanisms and mediators are exploited by tumors to avoid immune destruction. In an attempt to shed more light on the immunosuppressive environment in uterine tumors, we analyzed the presence of PD-L1, PD-L2, B7-H4, indoleamine 2,3-dioxygenase (IDO), galectin-1, galectin-3, arginase-1 activity and myeloid-derived suppressor cell (MDSC) infiltration. IDO, PD-L1, PD-L2 and B7-H4 were analyzed by immunohistochemistry. PD-L2 was mostly expressed at low levels in these tumors. We found high IDO expression in 21 % of endometrial carcinoma samples and in 14 % of uterine sarcoma samples. For PD-L1 and B7-H4, we found high expression in 92 and 90 % of endometrial cancers, respectively, and in 100 and 92 % of the sarcomas. Galectin-1 and 3 were analyzed in tissue lysates by ELISA, but we did not find an increase in both molecules in tumor lysates compared with benign tissues. We detected expression of galectin-3 by fibroblasts, immune cells and tumor cells in single-cell tumor suspensions. In addition, we noted a highly significant increase in arginase-1 activity in endometrial carcinomas compared with normal endometria, which was not the case for uterine sarcomas. Finally, we could demonstrate MDSC infiltration in fresh tumor suspensions from uterine tumors. These results indicate that the PD-1/PD-L1 interaction and B7-H4 could be possible targets for immune intervention in uterine cancer patients as well as mediation of MDSC function. These observations are another step toward the implementation of inhibitors of immunosuppression in the treatment of uterine cancer patients.

**Electronic supplementary material:**

The online version of this article (doi:10.1007/s00262-014-1537-8) contains supplementary material, which is available to authorized users.

## Introduction

Currently used immunotherapeutic regimens often result in immunological responses, such as antigen-specific T cell responses, but are all too often not accompanied by a correlating clinical response. One of the reasons for the lack of consistent clinical responses is thought to be attributable to immune escape exerted by the tumor. Various mechanisms of immune evasion by tumors have been described, but the picture in uterine tumors is still enigmatic. By increasing our knowledge on the involvement of these immune escape pathways in uterine tumors, we aim to identify new treatment options for this cancer type for which not much progress has been made in recent years.

One of the possible immune escape mechanisms are the so-called immune checkpoints. Immune checkpoints, some of which are briefly outlined below, comprise an array of pathways which the immune system occupies to maintain self-tolerance. Besides their natural function in the prevention of among others autoimmunity, these molecules also play an important role in antitumor immunity, more specifically in the prevention/blockage thereof. The two checkpoints most focused on are CTLA-4 and PD-1. Several inhibitors of these molecules are currently in clinical development and being analyzed in both the preclinical setting as well as in clinical trials, with the aim of improving treatment outcome and survival in cancer patients [1–4].

PD-L1 and PD-L2, both members of the B7-CD28 family, are ligands for the death receptor programmed death receptor 1 (PD-1), which play an important role in the central T cell tolerance during T cell development [5]. PD-L1 is expressed in a variety of tissues, such as placenta, heart and spleen cells as well as islets and leukocytes [5, 6]. In tumors, PD-L1 expression has been abundantly detected and is often associated with a poor prognosis [7–9]. The expression pattern of PD-L2 on the other hand is much more restricted. It is expressed mainly on dendritic cells and macrophages [6], but expression can be induced on several other immune and non-immune cells depending on environmental stimuli [10]. PD-L2 expression was shown in different tumor types, although mostly in a minority of patients [11–13]. PD-1/PD-L1 interactions have been shown to inhibit antigen-experienced T cells in the periphery, thereby protecting normal tissues from immune destruction [3]. PD-L2 on the other hand is regulated by Th2 cytokines and may itself possibly be involved in the modulation of Th2 responses [10].

B7-H4 mRNA is abundantly detected in human somatic tissues, but protein expression on tissues is limited [14]. On the contrary, B7-H4 protein expression has been found in many different types of human tumors [14], and soluble B7-H4 can be detected in the serum of cancer patients [15, 16]. B7-H4 has been shown to play a role in the inhibition of activation, proliferation and clonal expansion of both CD4^+^ and CD8^+^ T cells [14, 17].

Indoleamine 2,3-dioxygenase (IDO) is an intracellular enzyme mediating the rate-limiting step of tryptophan catabolism along the kynurenine pathway. IDO mediates “metabolic immune regulation” by depriving T cells of tryptophan and by the production of toxic metabolites, both leading to inhibition of T cell proliferation and induction of T cell death. Furthermore, IDO can convert naïve T cells into regulatory T cells (Treg) and enhances their activity [18]. IDO expression has been observed in a variety of cancers, and high IDO expression is associated with dismal prognosis [19].

Another family with important immunoregulatory functions is the galectin family. The current manuscript is focused on galectin-1 and galectin-3. Galectin-1 expression by tumors is associated with poor prognosis and the formation of metastasis through modulation of among others cell migration, adhesion and angiogenesis [20]. Besides its direct effects on tumor progression, galectin-1 also plays a role in tumor immune regulation by creating a bias toward a Th2 profile and activation of tolerogenic DC and IL-10 producing regulatory (Tr1) cells. Galectin-3 is involved in the differentiation and proliferation of several immune cells. It activates both lymphoid and myeloid cells, such as T cells, mast cells, monocytes and neutrophils [20]. Galectin-3 is also a negative regulator of immune cell function by controlling the anergic state of T cells [21]. Galectin-3 is capable of forming lattices with the T cell receptor complex, thereby intervening with T cell receptor signaling and subsequent T cell activation [20].

Other key players in tumor immune suppression are certain types of immune cells, such as Treg or myeloid-derived suppressor cells (MDSC). The latter are a heterogeneous population of immature myeloid cells with monocytic and/or granulocytic features. MDSC exert their suppressive function by depleting l-arginine from the tumor micro-environment via the production of arginase-1. The depletion of l-arginine subsequently causes a down-regulation of the ζ-chain of the T cell receptor, [22, 23] as well as cell cycle arrest of T cells [24]. Furthermore, MDSC produce reactive oxygen species (ROS) and nitric oxide (NO) radicals, which also capable of suppressing T cell function [25]. MDSC have been implicated in various tumors and are correlated with poor prognosis [26, 27]. MDSC levels might serve as a predictive marker for clinical outcome of oncologic treatment [25, 28].

We have used an immunohistochemical approach to validate the presence of IDO in uterine tumors, as well as to investigate the presence of PD-L1/PD-L2, to our knowledge not described in this type of tumor before, and B7-H4.

In addition, we have analyzed the expression of galectin-1 and 3 in uterine tissue samples by ELISA and confirmed galectin-3 expression on cellular subtypes in uterine tumor specimens by flow cytometry. The activity of arginase-1 in uterine tissue samples was determined, and we further provided evidence of MDSC infiltration in uterine tumor specimens by multi-parametric flow cytometry.

## Materials and methods

### Biopsy material

All biopsies were collected at the University Hospital Leuven, campus Gasthuisberg, from patients undergoing surgery at the Department of Gynecologic Oncology. When possible, multiple blocks were used from the same tumor to a maximum of three tumor blocks per tumor. The latter was done in order to obtain an idea of expression across the entire tumor. Samples of normal endometrium or myometrium were collected from patients undergoing surgery for benign afflictions.

The collection of all human samples was approved by the Institutional Review Board of the University Hospitals Leuven and written informed consent was obtained from all patients.

### Immunohistochemical staining

For all stainings, 4.0 μm formalin- or Bouin-fixed paraffin-embedded tissue slides were used. All slides were deparaffinized and rehydrated prior to staining. The general steps for each of the staining procedures are similar comprising the consecutive steps of endogenous alkaline phosphatase or peroxidase inhibition using 0.2 N HCl or 0.5 % H_2_O_2_ in methanol, respectively, followed by heat-induced epitope retrieval (HIER), blocking of slides to prevent aspecific antibody binding, and application of the appropriate antibodies and visualization methods. The specifications of these steps for each of the staining procedures are summarized in Table 1.

**Table 1:** Experimental conditions of IHC procedures

Target	IDO	PD-L1	PD-L2	B7-H4
*HIER* ^*a*^				
Buffer	Tris–HCl + 1 mM EDTA	Tris–HCl + 1 mM EDTA	Tris–HCl + 1 mM EDTA	Citrate
Time	2 h	1.5 h	2 h	1 h
Temperature	90 °C	90 °C	90 °C	90 °C
*Primary antibody*				
Antibody	Ms anti Hu IDO (Chemicon)	Rb anti Hu PD-L1 (Abcam)	Ms anti Hu PD-L2 (R&D Systems)	Rb anti Hu B7-H4 (Epitomics)
Final concentration	2 μg/ml	Ready to use	0.8 μg/ml	1/600 dilution
Secondary antibody	Go anti Ms IgG-Po + APAAP^b^ complex	Envision system anti Rb-HRP^c^	Envision system anti Ms-HRP	Envision system anti Rb-HRP
Visualization	NBT^d^	DAB^e^	DAB	DAB
Counterstaining	hematoxylin	hematoxylin	hematoxylin	hematoxylin

^a^ *HIER* heat-induced epitope retrieval

^b^ *APAAP* Alkaline phosphatase–anti-alkaline phosphatase

^c^ *HRP* horse radish peroxidase

^d^ *NBT* nitro blue tetrazolium

^e^ *DAB* 3,3′-diaminobenzidine

### Scoring system

Before scoring the tissue slides, tumor tissue was identified using a conventionally stained HE tissue slide of the same tumor. All slides were scored with a scoring system, partially adopted from Ino et al. [29]. All slides were given two separate scores. One for the percentage of total tumor cells showing expression, divided in three different categories: 1–25; 25–50; >50 %. Each of these parameters was subsequently given a score of 1, 2 and 3, respectively. The second score was given according to the intensity of the expression: 1 (weak), 2 (moderate) and 3 (strong). Depending on the sum of the two scores, a final value was given. A sum of 0–1 = final score 0; 2–3 = 1; 4–5 = 2 and 6 = 3. Based on these scores, a discrimination between biopsies with high and low expression levels was made, adopted from De Jong et al. [30]. Biopsies with a total score of 0–1 are classified as low score (lo), and biopsies with a total score of 2–3 are classified as high score (hi). The intensity scores which match the visual aspect of the staining were determined for each molecule separately.

### Tumor lysate preparation

150–200 mg of snap-frozen tissue samples were lysed by mechanical friction using magnetic stones after adding lysis buffer consisting of 250 mM Tris HCl, 750 mM NaCl, 0.5 % SDS, 2.5 % deoxycholic acid, 5 % Igepal and 0.01 % protease inhibitor cocktail (Mammalian Cell Lysis Kit; Sigma-Aldrich). After incubating the minced tissue material for 30 min at 4 °C, the lysed material was purified from debris through centrifugation. Protein content was determined using the Pierce BCA Protein Assay (Thermo Scientific) according to manufacturer’s instructions.

### Galectin-1 ELISA

Ninety-six-well plates were coated overnight with goat anti-human galectin-1 (R&D Systems, 2 μg/ml in PBS). After blocking the wells with 1 % BSA in PBS for 1 h at room temperature (RT), standards (recombinant galectin-1; range 25–0.39 ng/ml) and samples were added for 2 h at RT. Analyses were performed on tumor lysates at a concentration of 5 mg/ml. Detection was done using a combination of goat anti-human IgG (R&D systems, 200 ng/ml) and HRP-conjugated streptavidin, followed by incubation with TMB substrate. The reaction was stopped using 2 M H_2_SO_4_, and OD was measured at 450 nm, with 540 nm as reference wavelength.

### Galectin-3 ELISA

Galectin-3 detection was performed using a commercially available ELISA (R&D Systems) with minor modifications. For coating, mouse anti-human galectin-3 (2 μg/ml) was used. The galectin-3 standard was used within the range of 4,000–62.5 pg/ml. Detection was performed using mouse anti-human IgG in combination with HRP-conjugated streptavidin. All other steps are identical to galectin-1 detection.

### Arginase-1 activity assay

Measurement of arginase-1 activity, through determination of the urea content in tumor lysates (at a concentration of 5 mg/ml), was performed using the QuantiChrom™ Arginase Assay Kit (Bioassay Systems) following the manufacturer’s protocol.

### Preparation of single-cell suspensions from tumor biopsies

Single-cell suspensions of fresh tumor biopsies were prepared by a combination of mechanical dissociation and enzymatic digestion using the Human Tumor Dissociation Kit (Miltenyi Biotec) and the GentleMACS dissociator (Miltenyi Biotec). Briefly, the protocol for soft tumors was followed, according to manufacturer’s instructions. The cell suspensions were purified on a 40 μm cell strainer and counted with Türck’s solution. Single-cell suspensions from tumor biopsies were either used fresh or after cryopreservation for subsequent flow cytometric analysis.

### Flow cytometric analysis

Cell suspensions were first stained with a fixable viability dye (Fixable Viability Dye eFluor 506, eBioscience) for discrimination of live and dead cells. After washing with PBS, Fc receptor blocking was performed by adding a 10 % normal goat serum (Sigma-Aldrich) solution in PBS/0.5 % BSA. For single-cell tumor suspensions, red blood cell lysis was performed after the membrane staining using 1× PharmLyse (BD Pharmingen), according to manufacturer’s instructions.

Galectin-3 expression by primary tumor cell lines was evaluated by staining with galectin-3-PE (BioLegend) or the appropriate isotype control, either on the membrane or intracellularly after fixation/permeabilization using the FoxP3 Staining Buffer Set (eBioscience). Acquisition was performed with a FACS Canto II flow cytometer using BD FACS DIVA software, and 3 × 10^4^ cells were acquired per sample. Data analysis was done using BD FACS DIVA software. Cells were gated on FSC/SSC characteristics and analyzed for the expression of membranous or intracellular galectin-3.

For the analysis of galectin-3 expression by different cell types, we used the following antibody cocktail: CD90-FITC (BioLegend), galectin-3-PE (BioLegend), EpCAM-PerCP-Cy5.5 (BioLegend) and CD45-APC-H7 (BD Pharmingen). Intracellular expression of galectin-3 was assessed after fixation/permeabilization with the FoxP3 Staining Buffer Set (eBioscience). Acquisition was performed with a FACS Canto II flow cytometer using BD FACS DIVA software, and between 2.5 × 10^4^ and 1 × 10^6^ cells were acquired in the live gate per sample. Data analysis was done using BD FACS DIVA software. Cells were gated as follows: first, dead cells were gated out using the viability dye, and cells were gated based on FSC/SSC characteristics. Next, CD90^+^ fibroblasts, CD45^+^ immune cells and EpCAM^+^ tumor cells (for EMCAR), or CD45^−^ CD90^−^ tumor cells (for US) were gated, and each analyzed for the expression of membranous or intracellular galectin-3. Appropriate isotype controls were included to set the gates for the positive and negative populations.

For enumeration of MDSC, the following antibody cocktail was used: CD45-FITC (BioLegend), CD11b-PE (BioLegend), CD14-PerCP-Cy5.5 (BD Pharmingen), CD3-PE-Cy7 (BioLegend), CD19-PE-Cy7 (BioLegend), CD56-PE-Cy7 (BioLegend), CD15-APC (BioLegend), HLA-DR-APC-H7 (BD Pharmingen) and CD33-V450 (BD Horizon). Analysis of arginase-1 expression was done by replacing CD45-FITC with Arginase-1-fluorescein (R&D Systems) in the above-mentioned MDSC cocktail. For assessment of arginase-1 expression, cells were first stained for membrane markers, subsequently fixed and permeabilized using the FoxP3 Staining Buffer Set (eBioscience) and stained with arginase-1-fluorescein (R&D Systems). Acquisition was done as mentioned above for assessment of galectin-3 on tumor subpopulations. Data analysis was done using BD FACS DIVA software. The following gating strategy was used for enumeration of MDSC: first, dead cells were gated out using the viability dye, and cells were gated based on FSC/SSC characteristics, followed by CD45 positivity. Next, a gate was defined for lineage-negative (CD3-, CD19- and CD56-negative), HLA-DR^low/−^ cells. Within this population, CD11b^+^ CD14^−^ granulocytic MDSC and CD11b^+^ CD14^+^ monocytic MDSC were specified. On both cell types, the expression of CD15 and CD33 was documented. Arginase-1 expression by MDSC was analyzed as follows: dead cells were gated out using the viability dye, and cells were gated based on FSC/SSC characteristics. Next, the lineage-negative (CD3-, CD19- and CD56-negative), HLA-DR^low/−^ cell population was identified. Within this population, CD11b^+^ CD14^−^ granulocytic MDSC and CD11b^+^ CD14^+^ monocytic MDSC were defined, and the expression of arginase-1 on both subtypes was analyzed. Proper isotype controls were used to set the gates for the positive and negative populations.

### Statistics

Statistical analyses were done using the GraphPad Prism 5.0 software. All scoring data were analyzed using the nonparametric Kruskal–Wallis test, followed by the Dunn’s multiple comparison test. Differences in proportion were calculated using the Fischer exact test. Multiple group comparisons were performed using either ANOVA or Student’s *t* test. Comparison of survival curves was performed using the Log-Rank (Mantel–Cox) test.

## Results

### IDO expression in uterine tumors

**Fig. 1:**
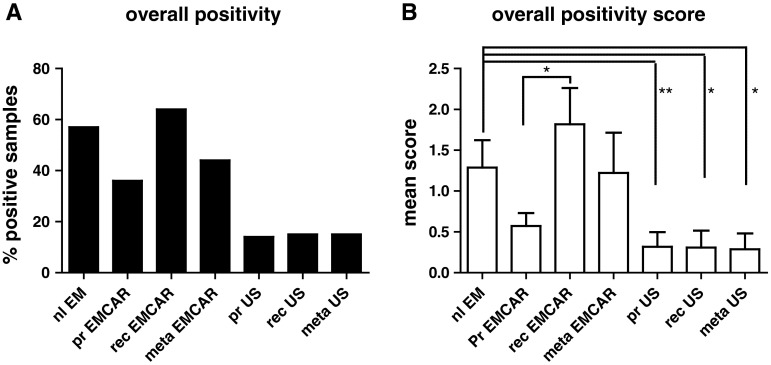
IDO expression levels in uterine tumors. **a** Percentage of tumors/normal endometrium positive for IDO. **b** Overall expression levels between groups

Patient characteristics of the IHC analysis of IDO, PD-L1, PD-L2 and B7-H4 are summarized in Supplementary Table 1. IDO is present in 57 % of normal endometria (Table 2). When looking at the carcinomatous tumors, we found IDO to be present in only 36 % of the primary endometrial cancers (EMCAR). It is slightly, yet not significantly, increased in the metastatic locations (44 %). Sixty percent of the currently analyzed recurrent EMCAR samples showed IDO expression. The enzyme is present in only a minority of sarcoma samples, and the expression level is generally low in this tumor type (Fig. 1). In comparison with normal endometrium, both the percentage of positive tumors (*p* < 0.01) and the expression level are significantly lower in primary US (*p* < 0.01) and recurrent US (*p* < 0.05). IDO is present in a significantly lower proportion of metastatic US lesions compared with normal endometrium (*p* < 0.05) and at a lower expression level (*p* < 0.05). The expression pattern of IDO was mainly cytoplasmic. In the EMCAR biopsies, in some tumors, clear apical expression could be detected (Supplemental Fig. 1). In addition, IDO expressing infiltrating cells could be detected in the tumor periphery of some of the analyzed tumors, both carcinoma and sarcoma.

**Table 2:** Immune checkpoint and IDO expression in different sample types

Sample	IDO		PD-L1		PD-L2		B7-H4	
	*N*	% positive	*N*	% positive	*N*	% positive	*N*	% positive
nl EM^a^	14	57	16	81	15	47	15	100
pr EMCAR^b^	28	36	29	83	30	40	30	100
rec EMCAR^c^	11	60	9	67	5	60	11	100
meta EMCAR^d^	9	44	9	100	9	44	10	90
pr US^e^	22	14	21	100	19	32	26	100
rec US^f^	13	15	11	100	4	0	13	92
meta US^g^	13	15	10	100	11	27	13	100

^a^ *nl EM* normal endometrium

^b^ *pr EMCAR* primary endometrial carcinoma

^c^ *rec EMCAR* recurrent endometrial carcinom

^d^ *meta EMCAR* metastatic endometrial carcinoma

^e^ *pr US* primary uterine sarcoma

^f^ *rec US* recurrent uterine sarcoma

^g^ *meta US* metastatic uterine sarcom

Twenty-one percent of primary EMCAR samples were IDO^hi^, while for US, 14 % of primary tumors were IDO^hi^, indicating that IDO blockade could be a useful strategy in a minority of patients.

### PD-L1 expression

**Fig. 2:**
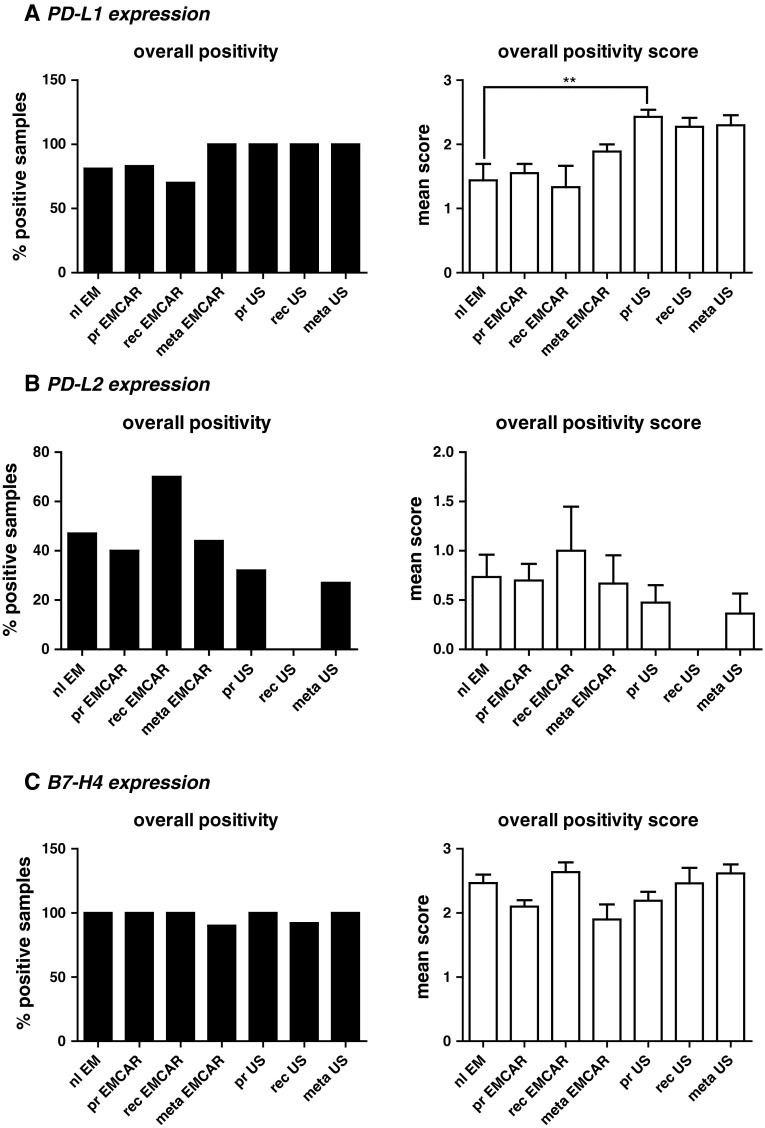
Immune checkpoints in uterine cancer. *Left panels* percentage of positive tumors/normal endometrium. *Right panels* Overall expression levels between groups. **a** IHC analysis of PD-L1 expression. **b** IHC analysis of PD-L2 expression. **c** IHC analysis of B7-H4 expression

Overall, PD-L1 is expressed in the vast majority of the currently tested samples (Fig. 2a). The expression pattern for PD-L1 was cytoplasmic in all of the analyzed biopsies (Supplemental figure 2). We found PD-L1 expression in 81 % of normal endometria. In 4/16 of the analyzed normal endometria, PD-L1^+^ cells infiltrating the endometrium were seen. PD-L1 expression in tumors was found in 70–80 % of the EMCAR samples and in all of the US samples. The number of analyzed samples and the corresponding percentages for each group are summarized in Table 2. When analyzing the expression level, we found an up-regulation in primary sarcomas in comparison with normal endometrium (*p* < 0.01). Overall, the expression level of PD-L1 was moderate to high. Of the patients with primary EMCAR, 72 % showed high PD-L1 expression, and for US, all patients were PD-L1^hi^, which shows that interference with the PD-1/PD-L1 axis could represent a promising new treatment option for uterine cancer patients. For EMCAR, we noticed a trend toward improved survival for patients with PD-L1^hi^ tumors (*p* = 0.084, data not shown).

### PD-L2 expression

The expression of PD-L2 was different from the expression of PD-L1 (Table 2). We found that 47 % of normal endometria expressed PD-L2. In EMCAR in general, the percentage of positive tumor samples varied from 40 to 70 %, while in the US group, this ranged from 0 to 32 % positive tumors. In general, the intensity scores for all the biopsies were low to moderate, and we did not find any differences in expression levels between any of the analyzed groups (Fig. 2b). Next to intracytoplasmic expression in tumor cells, we found the presence of PD-L2^+^ cells, possibly reflecting immune cell infiltrates in 10/30 (33 %) analyzed primary EMCAR biopsies as well as in 2/9 (22 %) of the included metastatic EMCAR lesions. The infiltrating cells were found intratumorally, in the tumoral stroma and in the tumor periphery (Supplemental figure 3).

Overall, in primary tumors, 30 % of EMCAR patients and 16 % of US patients showed high PD-L2 expression, thereby demonstrating the potential of PD-L2 blockade in a limited proportion of uterine cancer patients.

### B7-H4 expression

B7-H4 is expressed in the vast majority of the currently analyzed biopsies (Table 2). In-depth analysis of the expression levels revealed no differences between any of the groups (Fig. 2c). B7-H4 is expressed exclusively in the cytoplasm (Supplemental figure 4). Due to the visual aspects of the staining, discrimination of infiltrating cells was not possible.

The high expression levels of B7-H4 in both primary EMCAR (90 % of samples) and primary US (92 % of samples) make this an attractive candidate for uterine cancer therapy.

### Galectin-1 and galectin-3 levels

**Fig. 3:**
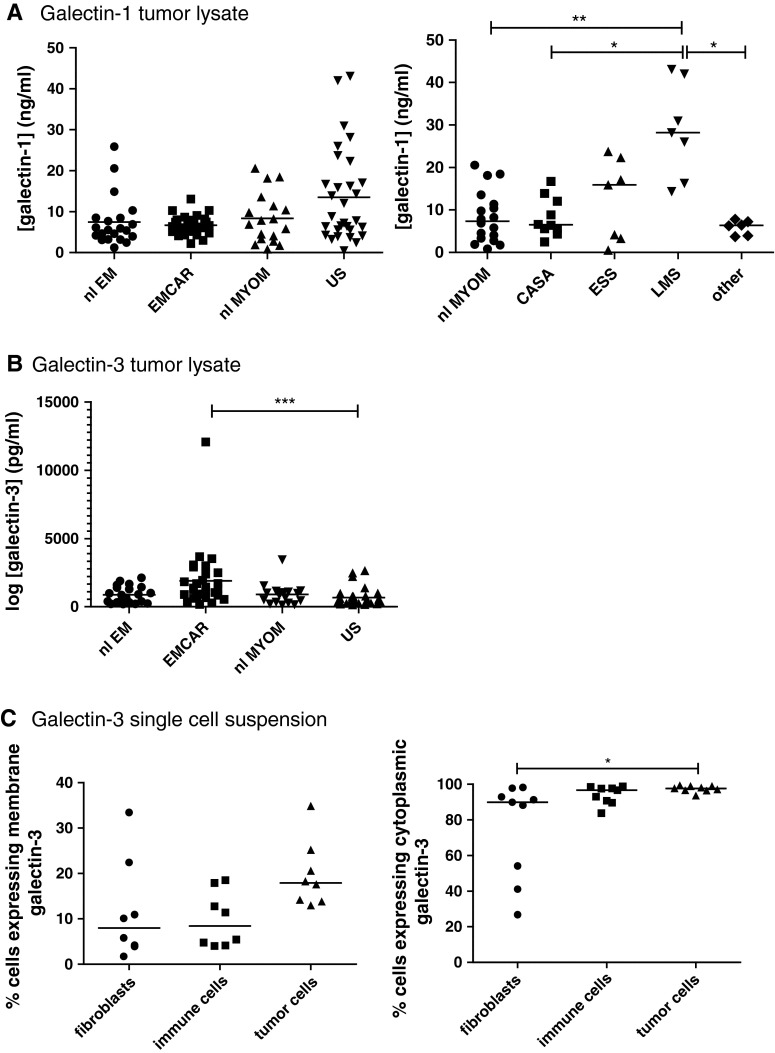
Galectin expression in patient plasma and uterine tissue samples. **a** galectin-1 expression in uterine tissue. *Left panel* overall galectin-1 expression. *Right panel* galectin-1 expression in myometrium versus sarcoma histological subtypes. **b** Galectin-3 expression in uterine tissue. **c** Flow cytometric detection of galectin-3 on different cell populations within single-cell tumor suspensions. *Left panel* membranous galectin-3 expression. *Right panel* intracellular galectin-3 expression

Galectin-1 and 3 presence in tumor tissue lysates was tested by ELISA in 29 EMCAR patients, 30 US patients, 21 normal/benign endometria and 18 normal myometria. The characteristics of the patients used for this study are summarized in Supplementary Table 2. We found that galectin-1 is present in all of the different analyzed tissues, without significant differences between the entire sample groups (Fig. 3a). When subdividing the US group, a significant galectin-1 up-regulation was found in leiomyosarcomas compared with myometrium (*p* < 0.01), carcinosarcomas (*p* < 0.05) and other US subtypes (*p* < 0.05). For galectin-3, we found a significantly higher intratumoral level in EMCAR samples compared with US samples (*p* < 0.05), shown in Fig. 3b.

For galectin-3, it was determined whether it is expressed on EMCAR and US cell lines (primary cell lines generated in our lab, kind gift from Dr. Coenegrachts) by flow cytometric analysis, and limited galectin-3 expression was detected on the plasma membrane, but nearly complete intracellular expression was found in these cell lines (Supplementary Table 3). Further assessment of its expression levels on fibroblasts, immune cells and tumor cells in single-cell suspensions from fresh tumor biopsies (tumor characteristics shown in Supplementary Table 4) firstly showed considerable variation in the cellular composition of the single-cell tumor suspensions: 1.24–16.16 % fibroblasts, 15.19–75.07 % immune cells and 6.73–80.21 % tumor cells (data not shown). When gating on these different cell populations, a slightly higher percentage of membranous galectin-3 positivity on tumor cells versus fibroblasts and immune cells was found, although this did not reach statistical significance, shown in Fig. 3c. With regard to intracellular galectin-3 expression, the majority of fibroblasts, immune cells and tumor cells expressed galectin-3. Intracellular galectin-3 expression was significantly higher in tumor cells versus fibroblasts (*p* < 0.05).

### Arginase-1 activity and MDSC infiltration

**Fig. 4:**
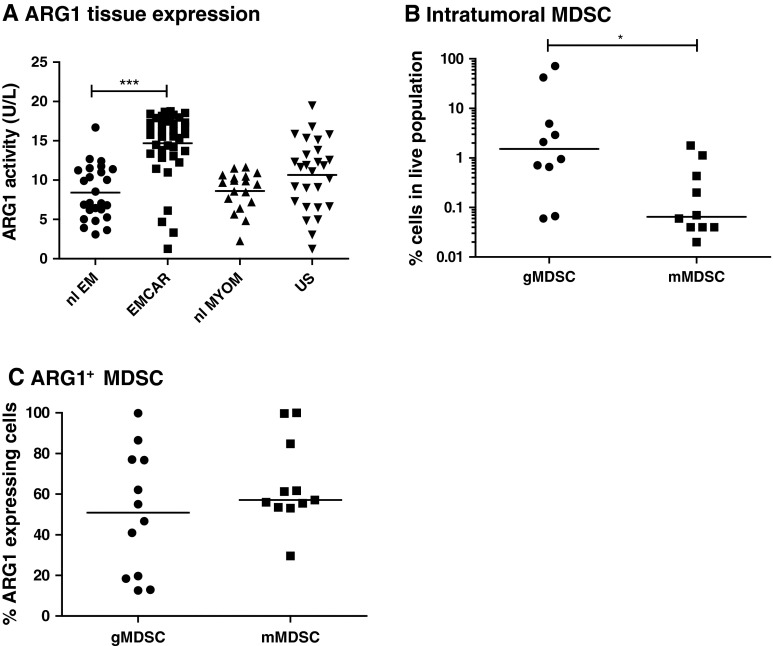
Arginase-1 activity in uterine tissues and MDSC infiltration in uterine tumors. **a** Arginase-1 activity in uterine tissues. **b** MDSC infiltration in uterine tumors. **c** Arginase-1 expression by MDSC infiltrating uterine tumors

Arginase-1 activity was measured in tissue lysates of 25 normal endometria, 39 EMCAR, 27 US and 18 normal myometria. Clinical parameters of all patient samples are depicted in Supplementary Table 5. Activity was significantly up-regulated in EMCAR samples compared with normal endometria (*p* < 0.0001). No difference in activity was found between normal myometria and US samples (Fig. 4a). These data suggested a possible implication of MDSC in endometrial carcinoma and inspired us to explore whether MDSC infiltration can be found in EMCAR and US tumors. Therefore, single-cell suspensions from fresh tumor biopsies were prepared (tumor characteristics shown in Supplementary Table 6), and flow cytometric analysis to detect MDSC and assess their arginase-1 expression was performed. MDSC infiltration was indeed detected in tumor specimens, and these cells were mainly of granulocytic phenotype (lin^−^ HLA-DR^lo/−^ CD11b^+^ CD14^−^; Fig. 4b). Tumors contained significantly more granulocytic compared with monocytic MDSC (*p* = 0.0124). However, both MDSC subtypes expressed similar arginase-1 levels (Fig. 4c). The majority of the granulocytic MDSC were CD15^+^, with approximately half of them co-expressing CD33. The monocytic MDSC were mainly CD15^+^ CD33^+^ (approximately 60 %), with an additional 24 % of CD15^+^ CD33^−^ cells (data not shown).

## Discussion

In the current research, we have evaluated the expression of PD-L1, PD-L2, B7-H4, IDO, galectin-1 and galectin-3 in uterine tumors and documented the arginase-1 activity and MDSC infiltration in these tumors. To our knowledge, this is the first paper to describe the presence of PD-L1 and PD-L2 in endometrial cancer as such, and we are not aware that any of the currently described molecules have been described in uterine sarcoma so far, except for possibly IDO [31], galectin-1 [32] and galectin-3 [32–34].

Both PD-1 ligands have previously been implicated in different types of cancer, although more is known about PD-L1 expression in several types of tumors, such as melanoma, brain tumors, lung cancer, urothelial cancer and pancreatic cancer [7, 35–38]. In soft tissue sarcomas in general, one study found expression of PD-L1 in 65 % of cases [9]. To date, little information is available on the expression of both ligands in gynecological tumors in general. Our data on PD-L1 expression show that it is expressed in the vast majority of both normal endometrial samples and of endometrial tumors, with no difference in proportion or expression level between any of the analyzed groups. In contrast to what we anticipated, we observed a trend toward improved overall survival in patients with PD-L1^hi^ tumors. However, this phenomenon has previously been described in melanoma, merkel cell carcinoma and MMR-proficient colorectal carcinoma [39–41]. Up-regulation of PD-L1 has been shown to be driven by IFNγ and CD8^+^ T cells. Hence, PD-L1 expression could be viewed as a result of an ongoing endogenous anti-tumor immune response and thus may represent a negative feedback loop dependent on an infiltrating immune response [42, 43].

PD-L2 has long been thought to be present only on dendritic cells and macrophages. However, more evidence is beginning to emerge of PD-L2 presence on somatic tissues and in cancer as well. It has, for example, been shown to be present in cancer-associated fibroblasts (Nazareth et al. [10]) as well as in esophageal [13] and hepatocellular carcinoma [11]. Most of the currently analyzed biopsies were negative for PD-L2, except in the recurrent carcinoma group. Similar to our results of low expression in the majority of tumors, a study in ovarian cancer has shown that the majority of the tumors are negative for PD-L2 [12]. Both ligands have also been investigated in cervical cancer [44], both being present in only a minority of the investigated samples.

B7-H4 has been shown in different tumors, summarized by He et al. [14]. Quite some data are available on its presence in breast cancer and ovarian cancer. It has been shown to be present intracellularly in ovarian tumor cells and on tumor-associated macrophages (TAM) in these tumors [45, 46]. B7-H4 expression levels were correlated with patient outcome [45]. Two studies report on B7-H4 expression in endometrial cancer [47, 48]. Both studies show B7-H4 expression in normal endometrium, and B7-H4 staining was shown to increase from normal to malignant endometrial glands. Our results are somewhat contradictory since we found high B7-H4 levels in almost all of the analyzed tissue samples in both benign and malignant endometrial tissues as well as in US samples. This observation might be due to the use of different antibodies, staining protocols and/or scoring protocols.

The immune inhibitory enzyme IDO has also been shown to play an important role in cancer-induced immunosuppression by depriving T cells of tryptophan and thus preventing their activation [49]. Uyttenhove et al. [31] described IDO in 25 different tumors. IDO has previously been detected in ovarian [31, 50] as well as endometrial tumors [29–31, 51, 52]. We have found high IDO expression in 21 % of primary endometrial tumors, which is in line with data reported by De Jong et al. (18,1 %), but lower than the results obtained by Ino et al. (46–49 %). We have also found an increased IDO expression level in recurrent EMCAR compared with primary EMCAR, pointing to increased immunosuppression in recurrent tumors. We found IDO expression only in a minority of US, in corroboration with Uyttenhove et al., who found IDO expression in 20 % of sarcomas. However, the exact subtype of sarcoma is not specified any further [31]. Except for PD-L1, no correlation between high and low expression and patient overall survival was found (data not shown). However, the currently analyzed sample population is too small to perform sound survival analyses. Based on the current data, no definitive conclusion can be made regarding patient survival. When analyzing the current sample population, no differences were found regarding FIGO stage, histological subtype or histological grade, except for a significantly lower expression level of IDO in grade 3 tumors compared with grade 1 (*p* < 0.05; data not shown). In addition, we could not find a difference in expression level between type I and type II EMCAR for any of the analyzed mediators. We do, however, acknowledge that the current sample sizes are too small to perform adequate statistical analyses regarding sub-parameters, which may bias or mask possible differences in expression levels.

Galectin-1 and galectin-3 have both been described in tumors. Galectin-1 and galectin-3 are both up-regulated in among others bladder carcinoma [53, 54], head and neck squamous cell carcinoma (HNSCC) [55] and ovarian cancer [56, 57]. Several studies have been published on galectins in endometrial cancer, with contradictory results. Galectin-1, but not galectin-3, has been shown to be increased in EMCAR compared with normal endometrium [58]. Similarly, galectin-1 was found to be increased in endometrioid EMCAR from well differentiated to undifferentiated carcinoma [59]. Contradictory to the data by Van den Brule et al. [58], Brustmann et al. [60] were able to show an increase in galectin-3 in EMCAR samples compared with normal endometrium samples. However, Ege et al. [61] reported decreased galectin-3 expression in EMCAR samples compared with normal endometrium. We currently found no increased galectin-1 or galectin-3 expression in tissue lysates of EMCAR or US compared with normal/benign controls. However, galectin-1 levels in lysates of leiomyosarcomas were significantly higher compared with myometrium, carcinosarcomas and other histological subtypes of US. We further showed both membranous and cytoplasmic expression of galectin-3 on single-cell tumor suspensions from primary tumors and on uterine cancer cell lines. In cancer, there is often a change in intracellular localization of galectin-3. Brustmann et al. [60] found an increased nuclear expression in EMCAR. Data of Mylonas et al. showed that it can be detected in both the cytoplasm and the nucleus [58], but exclusively, cytoplasmic expression was associated with increased myometrial invasion of EMCAR [58]. In the current study, we used tissue lysates or membrane/intracellular flow cytometric analysis, and consequently, no analysis on galectin location could be done to corroborate these data. Weissenbacher et al. [34] analyzed the expression of galectin-1 and galectin-3 in uterine leiomyosarcoma. Contradictory to what we currently found in the total sarcoma population for galectin-1, they found no difference in galectin-1 or galectin-3 expression when compared with normal myometrium. This discrepancy may in part be explained by the use of a semi-quantitative versus a quantitative analysis method in the current setting, which creates some difficulties for precise comparison of results.

Myeloid-derived suppressor cells have been shown to play an important role in cancer immunosuppression. de Boniface et al. [62] showed increased expression of arginase-1 on MDSC in breast cancer patients, which correlated with tumor grade. Arginase-releasing MDSC exerting detrimental effects on T cells are also increased in patient blood of renal cell carcinoma (RCC) patients [63, 64]. We have currently analyzed the activity of arginase-1 as a surrogate for MDSC. We found increased arginase-1 activity in EMCAR lysates compared with normal endometrium, indicating a possible involvement of MDSC in EMCAR. Furthermore, we showed for the first time that both EMCAR and US tumors contain MDSC infiltrates that express arginase-1.

In conclusion, we report the presence of the immune checkpoints PD-L1/PD-L2 and B7-H4 in uterine tumors as well as the presence of the immune inhibitory molecules IDO, arginase-1, galectin-1 and galectin-3 and the immunosuppressive MDSC. The current data in conjunction with positive results of currently ongoing clinical trials and preclinical studies with inhibitors for these molecules advocate for a possible future use in patients suffering from uterine tumors. In our opinion, IDO and PD-L2 are useful targets for immune inhibition in a minority of uterine cancer patients. Both PD-L1 and B7-H4 could represent targets in both tumor types, considering their high expression levels. Finally, intervention with MDSC function offers a new treatment perspective in EMCAR patients.

## Electronic supplementary material

Below is the link to the electronic supplementary material.

Supplementary material 1 (PDF 424 kb)

## Abbreviations

EMCAR: Endometrial carcinoma
IDO: Indoleamine 2,3-dioxygenase
MDSC: Myeloid-derived suppressor cell
PD-(L): Programmed death (ligand)
US: Uterine sarcoma

## References

[1] Pardoll DM (2012). The blockade of immune checkpoints in cancer immunotherapy. Nat Rev Cancer.

[2] Brahmer JR, Tykodi SS, Chow LQ, Hwu WJ, Topalian SL, Hwu P, Drake CG, Camacho LH, Kauh J, Odunsi K, Pitot HC, Hamid O, Bhatia S, Martins R, Eaton K, Chen S, Salay TM, Alaparthy S, Grosso JF, Korman AJ, Parker SM, Agrawal S, Goldberg SM, Pardoll DM, Gupta A, Wigginton JM (2012). Safety and activity of anti-PD-L1 antibody in patients with advanced cancer. N Engl J Med.

[3] Topalian SL, Drake CG, Pardoll DM (2012). Targeting the PD-1/B7-H1(PD-L1) pathway to activate anti-tumor immunity. Curr Opin Immunol.

[4] Topalian SL, Hodi FS, Brahmer JR, Gettinger SN, Smith DC, McDermott DF, Powderly JD, Carvajal RD, Sosman JA, Atkins MB, Leming PD, Spigel DR, Antonia SJ, Horn L, Drake CG, Pardoll DM, Chen L, Sharfman WH, Anders RA, Taube JM, McMiller TL, Xu H, Korman AJ, Jure-Kunkel M, Agrawal S, McDonald D, Kollia GD, Gupta A, Wigginton JM, Sznol M (2012). Safety, activity, and immune correlates of anti-PD-1 antibody in cancer. N Engl J Med.

[5] Blank C, Gajewski TF, Mackensen A (2005). Interaction of PD-L1 on tumor cells with PD-1 on tumor-specific T cells as a mechanism of immune evasion: implications for tumor immunotherapy. Cancer Immunol Immunother.

[6] Fife BT, Bluestone JA (2008). Control of peripheral T-cell tolerance and autoimmunity via the CTLA-4 and PD-1 pathways. Immunol Rev.

[7] Hino R, Kabashima K, Kato Y, Yagi H, Nakamura M, Honjo T, Okazaki T, Tokura Y (2010). Tumor cell expression of programmed cell death-1 ligand 1 is a prognostic factor for malignant melanoma. Cancer.

[8] Mu CY, Huang JA, Chen Y, Chen C, Zhang XG (2011). High expression of PD-L1 in lung cancer may contribute to poor prognosis and tumor cells immune escape through suppressing tumor infiltrating dendritic cells maturation. Med Oncol.

[9] Kim JR, Moon YJ, Kwon KS, Bae JS, Wagle S, Kim KM, Park HS, Lee H, Moon WS, Chung MJ, Kang MJ, Jang KY (2013). Tumor infiltrating PD1-positive lymphocytes and the expression of PD-L1 predict poor prognosis of soft tissue sarcomas. PLoS ONE.

[10] Rozali EN, Hato SV, Robinson BW, Lake RA, Lesterhuis WJ (2012) Programmed death ligand 2 in cancer-induced immune suppression. Clinical and Developmental Immunology. doi:10.1155/2012/656340 10.1155/2012/656340 PMC3350956 22611421

[11] Gao Q, Wang XY, Qiu SJ, Yamato I, Sho M, Nakajima Y, Zhou J, Li BZ, Shi YH, Xiao YS, Xu Y, Fan J (2009). Overexpression of PD-L1 significantly associates with tumor aggressiveness and postoperative recurrence in human hepatocellular carcinoma. Clin Cancer Res.

[12] Hamanishi J, Mandai M, Iwasaki M, Okazaki T, Tanaka Y, Yamaguchi K, Higuchi T, Yagi H, Takakura K, Minato N, Honjo T, Fujii S (2007). Programmed cell death 1 ligand 1 and tumor-infiltrating CD8+ T lymphocytes are prognostic factors of human ovarian cancer. Proc Natl Acad Sci USA.

[13] Ohigashi Y, Sho M, Yamada Y, Tsurui Y, Hamada K, Ikeda N, Mizuno T, Yoriki R, Kashizuka H, Yane K, Tsushima F, Otsuki N, Yagita H, Azuma M, Nakajima Y (2005). Clinical significance of programmed death-1 ligand-1 and programmed death-1 ligand-2 expression in human esophageal cancer. Clin Cancer Res.

[14] He C, Qiao H, Jiang H, Sun X (2011) The inhibitory role of b7-h4 in antitumor immunity: association with cancer progression and survival. Clinical and Developmental Immunology. doi:10.1155/2011/695834 10.1155/2011/695834 PMC3195678 22013483

[15] Oikonomopoulou K, Li L, Zheng Y, Simon I, Wolfert RL, Valik D, Nekulova M, Simickova M, Frgala T, Diamandis EP (2008). Prediction of ovarian cancer prognosis and response to chemotherapy by a serum-based multiparametric biomarker panel. Br J Cancer.

[16] Simon I, Katsaros D, Rigault de la Longrais I, Massobrio M, Scorilas A, Kim NW, Sarno MJ, Wolfert RL, Diamandis EP (2007). B7-H4 is over-expressed in early-stage ovarian cancer and is independent of CA125 expression. Gynecol Oncol.

[17] Yi KH, Chen L (2009). Fine tuning the immune response through B7-H3 and B7-H4. Immunol Rev.

[18] Munn DH (2012). Blocking IDO activity to enhance anti-tumor immunity. Front Biosci.

[19] Munn DH, Mellor AL (2013). Indoleamine 2,3 dioxygenase and metabolic control of immune responses. Trends Immunol.

[20] Yang RY, Rabinovich GA, Liu FT (2008). Galectins: structure, function and therapeutic potential. Expert Rev Mol Med.

[21] Demotte N, Wieers G, Van Der Smissen P, Moser M, Schmidt C, Thielemans K, Squifflet JL, Weynand B, Carrasco J, Lurquin C, Courtoy PJ, van der Bruggen P (2010). A galectin-3 ligand corrects the impaired function of human CD4 and CD8 tumor-infiltrating lymphocytes and favors tumor rejection in mice. Cancer Res.

[22] Munder M (2009). Arginase: an emerging key player in the mammalian immune system. Br J Pharmacol.

[23] Rodriguez PC, Quiceno DG, Zabaleta J, Ortiz B, Zea AH, Piazuelo MB, Delgado A, Correa P, Brayer J, Sotomayor EM, Antonia S, Ochoa JB, Ochoa AC (2004). Arginase I production in the tumor microenvironment by mature myeloid cells inhibits T-cell receptor expression and antigen-specific T-cell responses. Cancer Res.

[24] Rodriguez PC, Quiceno DG, Ochoa AC (2007). L-arginine availability regulates T-lymphocyte cell-cycle progression. Blood.

[25] Nagaraj S, Gabrilovich DI (2010). Myeloid-derived suppressor cells in human cancer. Cancer J.

[26] Huang A, Zhang B, Wang B, Zhang F, Fan KX, Guo YJ (2013). Increased CD14(+)HLA-DR (−/low) myeloid-derived suppressor cells correlate with extrathoracic metastasis and poor response to chemotherapy in non-small cell lung cancer patients. Cancer Immunol Immunother.

[27] Meyer C, Cagnon L, Costa-Nunes CM, Baumgaertner P, Montandon N, Leyvraz L, Michielin O, Romano E, Speiser DE (2013). Frequencies of circulating MDSC correlate with clinical outcome of melanoma patients treated with ipilimumab. Cancer Immunol Immunother.

[28] Walter S, Weinschenk T, Stenzl A, Zdrojowy R, Pluzanska A, Szczylik C, Staehler M, Brugger W, Dietrich PY, Mendrzyk R, Hilf N, Schoor O, Fritsche J, Mahr A, Maurer D, Vass V, Trautwein C, Lewandrowski P, Flohr C, Pohla H, Stanczak JJ, Bronte V, Mandruzzato S, Biedermann T, Pawelec G, Derhovanessian E, Yamagishi H, Miki T, Hongo F, Takaha N, Hirakawa K, Tanaka H, Stevanovic S, Frisch J, Mayer-Mokler A, Kirner A, Rammensee HG, Reinhardt C, Singh-Jasuja H (2012). Multipeptide immune response to cancer vaccine IMA901 after single-dose cyclophosphamide associates with longer patient survival. Nat Med.

[29] Ino K, Yoshida N, Kajiyama H, Shibata K, Yamamoto E, Kidokoro K, Takahashi N, Terauchi M, Nawa A, Nomura S, Nagasaka T, Takikawa O, Kikkawa F (2006). Indoleamine 2,3-dioxygenase is a novel prognostic indicator for endometrial cancer. Br J Cancer.

[30] de Jong RA, Kema IP, Boerma A, Boezen HM, der Want JJ, Gooden MJ, Hollema H, Nijman HW (2012). Prognostic role of indoleamine 2,3-dioxygenase in endometrial carcinoma. Gynecol Oncol.

[31] Uyttenhove C, Pilotte L, Theate I, Stroobant V, Colau D, Parmentier N, Boon T, Van den Eynde BJ (2003). Evidence for a tumoral immune resistance mechanism based on tryptophan degradation by indoleamine 2,3-dioxygenase. Nat Med.

[32] Schwarz G, Remmelink M, Decaestecker C, Gielen I, Budel V, Burchert M, Darro F, Danguy A, Gabius HJ, Salmon I, Kiss R (1999). Galectin fingerprinting in tumor diagnosis. Differential expression of galectin-3 and galectin-3 binding sites, but not galectin-1, in benign vs malignant uterine smooth muscle tumors. Am J Clin Pathol.

[33] Weissenbacher T, Vrekoussis T, Roeder D, Makrigiannakis A, Mayr D, Ditsch N, Friese K, Jeschke U, Dian D (2013). Analysis of epithelial growth factor-receptor (EGFR) phosphorylation in uterine smooth muscle tumors: correlation to mucin-1 and galectin-3 expression. Int J Mol Sci.

[34] Weissenbacher T, Kuhn C, Mayr D, Pavlik R, Friese K, Scholz C, Jeschke U, Ditsch N, Dian D (2011). Expression of mucin-1, galectin-1 and galectin-3 in human leiomyosarcoma in comparison to leiomyoma and myometrium. Anticancer Res.

[35] Inman BA, Sebo TJ, Frigola X, Dong H, Bergstralh EJ, Frank I, Fradet Y, Lacombe L, Kwon ED (2007). PD-L1 (B7-H1) expression by urothelial carcinoma of the bladder and BCG-induced granulomata: associations with localized stage progression. Cancer.

[36] Jacobs JF, Idema AJ, Bol KF, Nierkens S, Grauer OM, Wesseling P, Grotenhuis JA, Hoogerbrugge PM, de Vries IJ, Adema GJ (2009). Regulatory T cells and the PD-L1/PD-1 pathway mediate immune suppression in malignant human brain tumors. Neuro Oncol.

[37] Konishi J, Yamazaki K, Azuma M, Kinoshita I, Dosaka-Akita H, Nishimura M (2004). B7-H1 expression on non-small cell lung cancer cells and its relationship with tumor-infiltrating lymphocytes and their PD-1 expression. Clin Cancer Res.

[38] Zhang Y, Huang S, Gong D, Qin Y, Shen Q (2010). Programmed death-1 upregulation is correlated with dysfunction of tumor-infiltrating CD8 + T lymphocytes in human non-small cell lung cancer. Cell Mol Immunol.

[39] Taube JM, Anders RA, Young GD, Xu HY, Sharma R, McMiller TL, Chen SM, Klein AP, Pardoll DM, Topalian SL, Chen LP (2012). Colocalization of inflammatory response with B7-H1 expression in human melanocytic lesions supports an adaptive resistance mechanism of immune escape. Sci Transl Med.

[40] Droeser RA, Hirt C, Viehl CT, Frey DM, Nebiker C, Huber X, Zlobec I, Eppenberger-Castori S, Tzankov A, Rosso R, Zuber M, Muraro MG, Amicarella F, Cremonesi E, Heberer M, Iezzi G, Lugli A, Terracciano L, Sconocchia G, Oertli D, Spagnoli GC, Tornillo L (2013). Clinical impact of programmed cell death ligand 1 expression in colorectal cancer. Eur J Cancer.

[41] Lipson EJ, Vincent JG, Loyo M, Kagohara LT, Luber BS, Wang H, Xu H, Nayar SK, Wang TS, Sidransky D, Anders RA, Topalian SL, Taube JM (2013). PD-L1 expression in the merkel cell carcinoma microenvironment: association with inflammation, merkel cell polyomavirus, and overall survival. Cancer Immunol Res.

[42] Spranger S, Spaapen RM, Zha YY, Williams J, Meng YR, Ha TT, Gajewski TF (2013). Up-regulation of PD-L1, IDO, and T-regs in the melanoma tumor microenvironment is driven by CD8(+) T cells. Sci Transl Med.

[43] Lyford-Pike S, Peng SW, Young GD, Taube JM, Westra WH, Akpeng B, Bruno TC, Richmon JD, Wang H, Bishop JA, Chen LP, Drake CG, Topalian SL, Pardoll DM, Pai SI (2013). Evidence for a Role of the PD-1:PD-L1 Pathway in Immune Resistance of HPV-Associated Head and Neck Squamous Cell Carcinoma. Cancer Res.

[44] Karim R, Jordanova ES, Piersma SJ, Kenter GG, Chen L, Boer JM, Melief CJ, van der Burg SH (2009). Tumor-expressed B7-H1 and B7-DC in relation to PD-1 + T-cell infiltration and survival of patients with cervical carcinoma. Clin Cancer Res.

[45] Kryczek I, Wei S, Zhu G, Myers L, Mottram P, Cheng P, Chen L, Coukos G, Zou W (2007). Relationship between B7-H4, regulatory T cells, and patient outcome in human ovarian carcinoma. Cancer Res.

[46] Kryczek I, Zou L, Rodriguez P, Zhu G, Wei S, Mottram P, Brumlik M, Cheng P, Curiel T, Myers L, Lackner A, Alvarez X, Ochoa A, Chen L, Zou W (2006). B7-H4 expression identifies a novel suppressive macrophage population in human ovarian carcinoma. J Exp Med.

[47] Miyatake T, Tringler B, Liu W, Liu SH, Papkoff J, Enomoto T, Torkko KC, Dehn DL, Swisher A, Shroyer KR (2007). B7-H4 (DD-O110) is overexpressed in high risk uterine endometrioid adenocarcinomas and inversely correlated with tumor T-cell infiltration. Gynecol Oncol.

[48] Qian Y, Shen L, Cheng LF, Wu ZG, Yao HP (2011). B7-H4 expression in various tumors determined using a novel developed monoclonal antibody. Clin Exp Med.

[49] Prendergast GC (2008). Immune escape as a fundamental trait of cancer: focus on IDO. Oncogene.

[50] Okamoto A, Nikaido T, Ochiai K, Takakura S, Saito M, Aoki Y, Ishii N, Yanaihara N, Yamada K, Takikawa O, Kawaguchi R, Isonishi S, Tanaka T, Urashima M (2005). Indoleamine 2,3-dioxygenase serves as a marker of poor prognosis in gene expression profiles of serous ovarian cancer cells. Clin Cancer Res.

[51] Ino K, Yamamoto E, Shibata K, Kajiyama H, Yoshida N, Terauchi M, Nawa A, Nagasaka T, Takikawa O, Kikkawa F (2008). Inverse correlation between tumoral indoleamine 2,3-dioxygenase expression and tumor-infiltrating lymphocytes in endometrial cancer: its association with disease progression and survival. Clin Cancer Res.

[52] Sedlmayr P, Semlitsch M, Gebru G, Karpf E, Reich O, Tang T, Wintersteiger R, Takikawa O, Dohr G (2003). Expression of indoleamine 2,3-dioxygenase in carcinoma of human endometrium and uterine cervix. Adv Exp Med Biol.

[53] Cindolo L, Benvenuto G, Salvatore P, Pero R, Salvatore G, Mirone V, Prezioso D, Altieri V, Bruni CB, Chiariotti L (1999). Galectin-1 and galectin-3 expression in human bladder transitional-cell carcinomas. Int J Cancer.

[54] Demydenko D, Berest I (2009). Expression of galectin-1 in malignant tumors. Exp Oncol.

[55] Saussez S, Lorfevre F, Lequeux T, Laurent G, Chantrain G, Vertongen F, Toubeau G, Decaestecker C, Kiss R (2008). The determination of the levels of circulating galectin-1 and -3 in HNSCC patients could be used to monitor tumor progression and/or responses to therapy. Oral Oncol.

[56] Kim MK, Sung CO, Do IG, Jeon HK, Song TJ, Park HS, Lee YY, Kim BG, Lee JW, Bae DS (2011). Overexpression of Galectin-3 and its clinical significance in ovarian carcinoma. Int J Clin Oncol.

[57] van den Brule F, Califice S, Garnier F, Fernandez PL, Berchuck A, Castronovo V (2003). Galectin-1 accumulation in the ovary carcinoma peritumoral stroma is induced by ovary carcinoma cells and affects both cancer cell proliferation and adhesion to laminin-1 and fibronectin. Lab Investig.

[58] van den Brule FA, Buicu C, Berchuck A, Bast RC, Deprez M, Liu FT, Cooper DN, Pieters C, Sobel ME, Castronovo V (1996). Expression of the 67-kD laminin receptor, galectin-1, and galectin-3 in advanced human uterine adenocarcinoma. Hum Pathol.

[59] Mylonas I, Mayr D, Walzel H, Shabani N, Dian D, Kuhn C, Kunze S, Jeschke U, Friese K (2007). Mucin 1, Thomsen–Friedenreich expression and galectin-1 binding in endometrioid adenocarcinoma: an immunohistochemical analysis. Anticancer Res.

[60] Brustmann H, Riss D, Naude S (2003). Galectin-3 expression in normal, hyperplastic, and neoplastic endometrial tissues. Pathol Res Pract.

[61] Ege CB, Akbulut M, Zekioglu O, Ozdemir N (2011). Investigation of galectin-3 and heparanase in endometrioid and serous carcinomas of the endometrium and correlation with known predictors of survival. Arch Gynecol Obstet.

[62] de Boniface J, Mao Y, Schmidt-Mende J, Kiessling R, Poschke I (2012). Expression patterns of the immunomodulatory enzyme arginase 1 in blood, lymph nodes and tumor tissue of early-stage breast cancer patients. Oncoimmunology.

[63] Rodriguez PC, Ernstoff MS, Hernandez C, Atkins M, Zabaleta J, Sierra R, Ochoa AC (2009). Arginase I-producing myeloid-derived suppressor cells in renal cell carcinoma are a subpopulation of activated granulocytes. Cancer Res.

[64] Zea AH, Rodriguez PC, Atkins MB, Hernandez C, Signoretti S, Zabaleta J, McDermott D, Quiceno D, Youmans A, O’Neill A, Mier J, Ochoa AC (2005). Arginase-producing myeloid suppressor cells in renal cell carcinoma patients: a mechanism of tumor evasion. Cancer Res.

