# Supplementary material for: Mapping the immunosuppressive environment in uterine tumors: implications for immunotherapy
Source: Cancer Immunol Immunother. 2014 Mar 22;63(6):545–57. doi: 10.1007/s00262-014-1537-8 (PMC4024136; doi:10.1007/s00262-014-1537-8)
Supplement: Supplementary file 1 — Supplementary material 1 (PDF 424 kb) [file 262_2014_1537_MOESM1_ESM.pdf]

Supplementary Table 1: Clinical characteristics of biopsies used for IHC detection.

Patient characteristics	EMCAR[a] patients	US[b] patients
Total	48	47
Age (years)		
Mean	69,18	60,7
Range	43-86	26-85
Type		
Primary	27 (55,1%)	25 (53,2%)
Recurrence	11 (22,9%)	10 (21,3%)
Metastasis	7 (14,3%)	10 (21,3%)
Primary + metastasis	3 (6,1%)	1 (2,1%)
Metastasis + recurrence	0 (0,0%)	1 (2,1%)
FIGO stage[c] ,		
I	10 (33,3%)	
II	4 (13,3%)	
III	7 (23,3%)	
IV	9 (30%)	
Differentiation grade		
Low	7 (14,3%)	
Moderate	3 (6,1%)	
High	29 (59,2%)	
unknown	9 (18,75%)	
Histologic subtype		
Endometrioid	16 (32,7%)	
Clear cell	8 (16,3%)	
Serous papillary	20 (40,8%)	
Mixed	2 (4,16%)	
Carcinosarcoma	2 (4,08%)[d]	18 (38,3%)
Leiomyosarcoma		12 (25,5%)
Rhabdomyosarcoma		6 (12,8%)
Endometrial stromal sarcoma		7 (14,9%)
Adenosarcoma		4 (8,5%)

[a] EMCAR: endometrial carcinoma
[b] US: uterine sarcoma
[c] FIGO stage is given only for primary endometrial carcinoma
[d] Only carcinomatous tumor part included

Supplementary Table 2: Clinical characteristics of tissue lysates used for galectin detection.

Patient characteristics	EMCAR patients[e]	US patients[f]
Total	29	30
Age (years)		
Mean	67,1	61
Range	48-86	29-83
FIGO stage (2009)		
I	9 (31,0%)	
II	8 (27,6%)	
III	8 (27,6%)	
IV	4 (13,8%)	
Differentiation grade (primary tumor)		
Low	10 (34,5%)	
Moderate	6 (20,7%)	
High	13 (44,8%)	
Histologic subtype (primary tumor)		
Endometrioid	22 (75,9%)	
Clear cell	3 (10,35%)	
Serous papillary	3 (10,35%)	
Mixed	1 (3,4%)	
Carcinosarcoma		10 (33,3%)
Leiomyosarcoma		8 (26,6%)
Rhabdomyosarcoma		3 (10%)
Endometrial stromal sarcoma		7 (23,3%)
Unknown		1 (3,3%)
Ewing sarcoma		1 (3,3%)

[e] EMCAR: endometrial carcinoma
[f] US: uterine sarcoma

Supplementary Table 3: Galectin-3 expression in primary cancer cell lines

Cell line	Histology	Grade	FIGO stage	% positive cells	
				gal-3-M[g]	gal-3-C[h]
PC-EM-002	Endometrioid	2	II	0.54	85
PC-EM-005	Endometrioid	3	II	3	98
PC-EM-012	Endometrioid	2	IV	8	60
PC-EM-018	Mixed	3	Ia	0.81	98
PC-EM-033	Endometrioid	3	Ia	1	99
PC-EM-046	Endometrioid	3	Ib	5	80
PC-EM-076	Endometrioid	2	Ia	1,2	99,8
PC-SAR-25	Embryonal rhabdomyosarcoma	NA[i]	unknown	0,3	99,1

[g] gal-3-M: membranous expression
[h] gal-3-C: cytoplasmic expression
[i] NA: not applicable

Supplementary Table 4: Characteristics of tumors used for flow cytometric assessment of galectin-3

ID	Type	FIGO stage	Histology	Grade	Time point
13	EMCAR	IIIc	serous	3	recurrence
17	US	IV	leiomyosarcoma	3	primary
18	EMCAR	Ia	endometrioid	1	primary
19	EMCAR	Ia	endometrioid	1	primary
22	US	Ib	leiomyosarcoma	3	recurrence
24	EMCAR	Ia	endometrioid	2	primary
25	EMCAR	II	endometrioid	1/2	primary
26	EMCAR	Ia	endometrioid	1	primary
27	EMCAR	Ib	endometrioid	3	primary

Supplementary Table 5: Clinical characteristics of tissue lysates used for arginase-1 activity detection.

Characteristics	EMCAR[j] patients	US[k] patients
Total	39	27
Age (years)		
Mean	67,2	61
Range	36-86	29-83
FIGO stage		
I	10 (25,64%)	
II	10 (25,64%)	
III	9 (23,08%)	
IV	10 (25,64%)	
Differentiation grade		
Low	8 (20,51%)	
Moderate	4 (10,26%)	
High	17 (43,59%)	
Unknown	10 (25,64%)	
Histologic subtype (primary tumor)		
Endometrioid	20 (51,3%)	
Clear cell	8 (12,8%)	
Serous papillary	11 (28,2%)	
Mixed	3 (7,7%)	
Carcinosarcoma		9 (33,3%)
Leiomyosarcoma		8 (29,6%)
Rhabdomyosarcoma		2 (7,4%)
Endometrial stromal sarcoma		6 (22,2%)
Unknown		1 (3,75%)
Ewing sarcoma		1 (3,75%)

[j] EMCAR: endometrial carcinoma
[k] US: uterine sarcoma

Supplementary Table 6: Characteristics of tumors used for MDSC infiltration and arginase-1 expression by MDSC

ID	Type	FIGO stage	Histology	Grade	Time point	MDSC frequency	MDSC arginase-1
5	EMCAR	Ia	endometrioid	1	primary	yes	no
9	EMCAR	IIIa	mixed serous-clear cell	3	primary	yes	no
10	EMCAR	Ib	endometrioid	1	primary	yes	yes
11	EMCAR	Ia	endometrioid	1	primary	yes	yes
14	EMCAR	Ib-II	endometrioid	3	primary	yes	yes
17	US	IV	leiomyosarcoma	3	primary	no	yes
18	EMCAR	Ia	endometrioid	1	primary	no	yes
19	EMCAR	Ia	endometrioid	1	primary	no	yes
22	US	Ib	leiomyosarcoma	3	recurrence	no	yes
24	EMCAR	Ia	endometrioid	2	primary	yes	yes
25	EMCAR	II	endometrioid	1/2	primary	yes	yes
26	EMCAR	Ia	endometrioid	1	primary	yes	yes
27	EMCAR	Ib	endometrioid	3	primary	yes	yes
28	US	IV	myxoid		primary	yes	yes

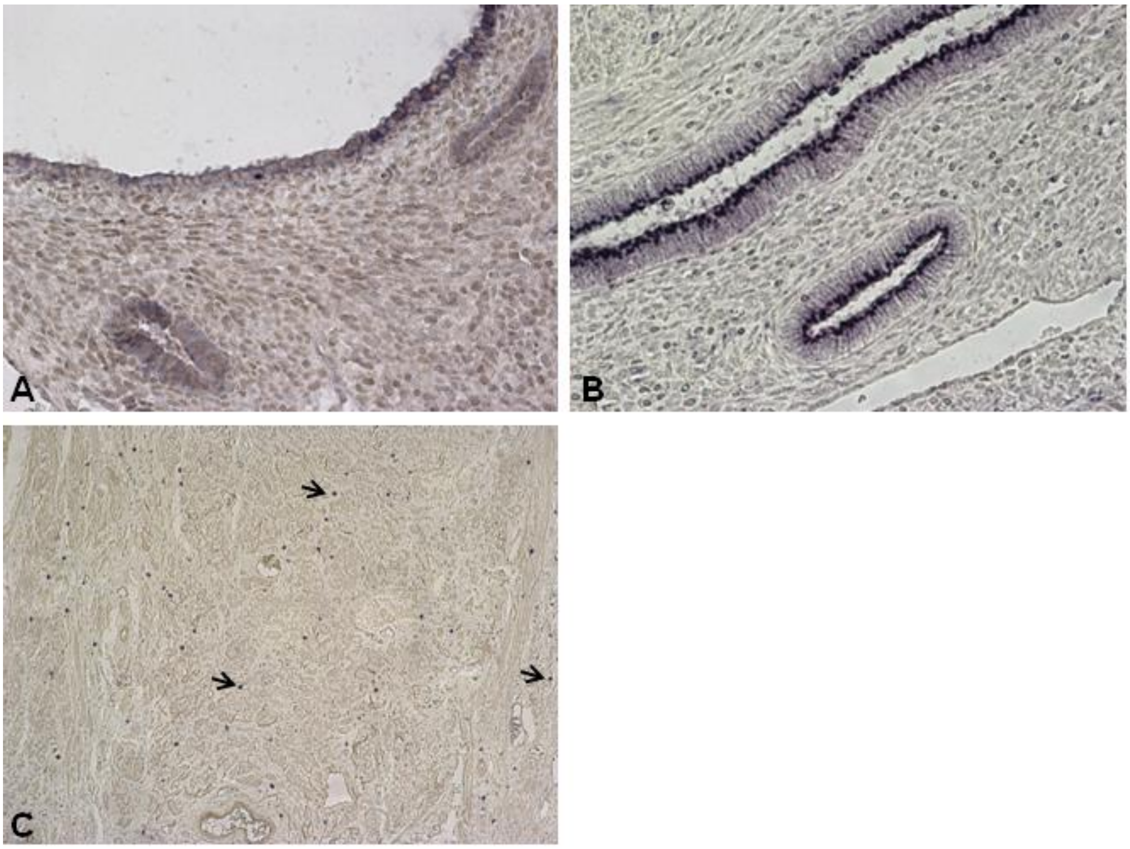

Supplemental Figure 1: IDO expression in endometrial biopsies. A. normal endometrium with moderate (intensity score 2) expression levels of IDO (40x enlarged). B. normal endometrium with clear apical IDO expression at high expression level (intensity score 3, 40x enlarged). C. endometrial carcinoma biopsy with numerous infiltrating cells in the tumor stroma (black arrows, 10x enlarged).

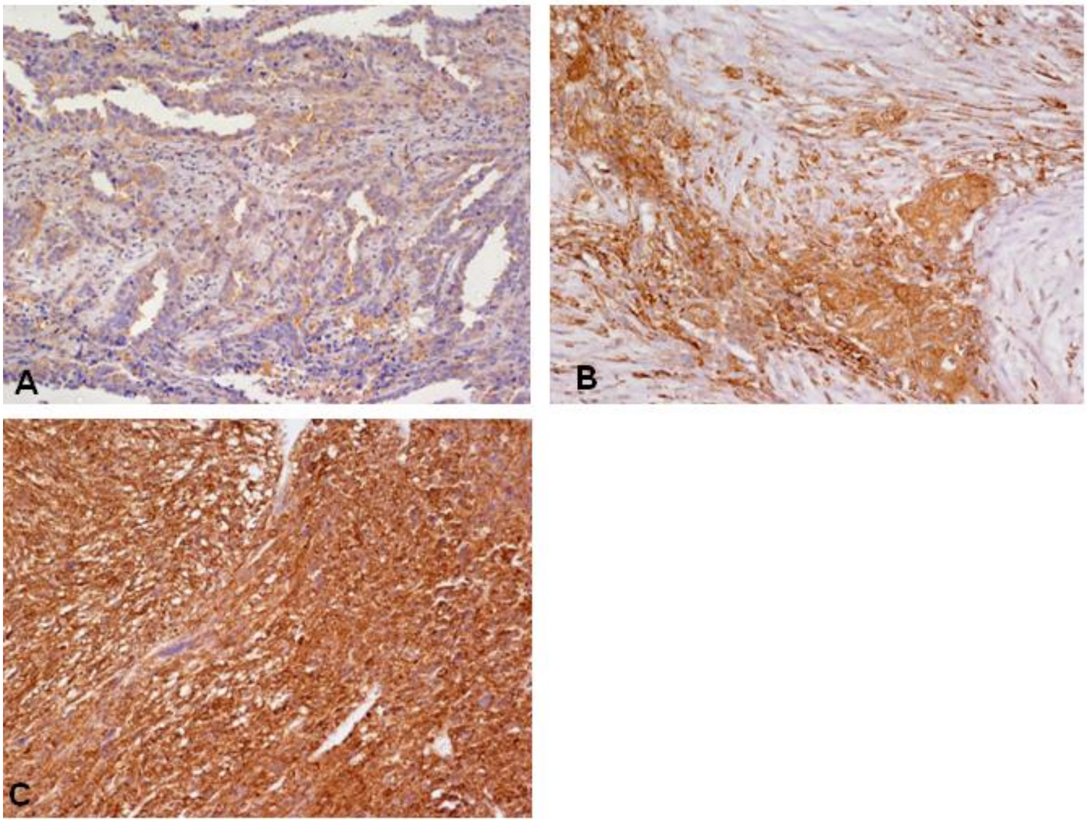

Supplemental Figure 2: PD-L1 expression in endometrial biopsies. A. endometrial carcinoma biopsy showing weak expression (intensity score 1, 20x enlarged). B. endometrial carcinoma expressing moderate (intensity score 2) PD-L1 levels (20x enlarged). C. high PD-L1 expression levels in uterine sarcoma (20x enlarged). High expression can clearly be seen on the cell membrane (top of picture).

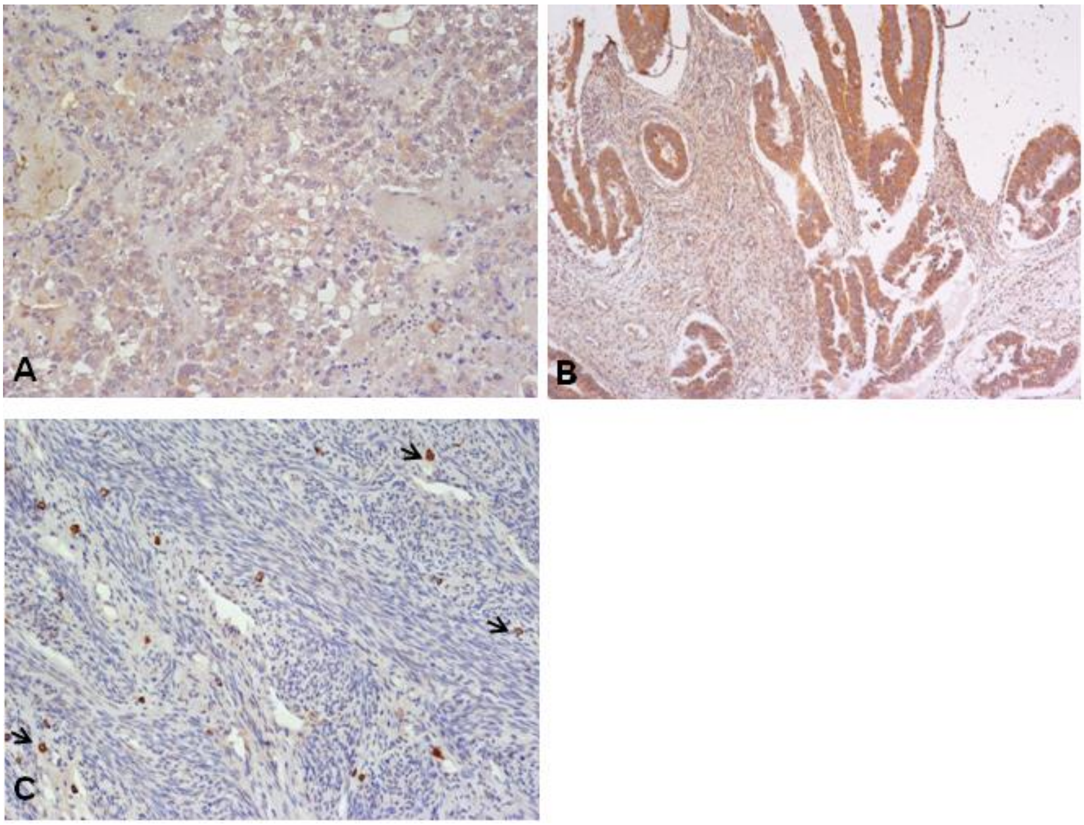

Supplemental Figure 3: PD-L2 expression in endometrial biopsies. Endometrial carcinoma samples showing A. weak expression (20x enlarged) and B. moderate expression level (10x enlarged) of PD-L2. C. representative biopsy showing the presence of PD-L2 positive cells infiltrating the tumor periphery, possibly reflecting the presence of immune cells (20x enlarged).

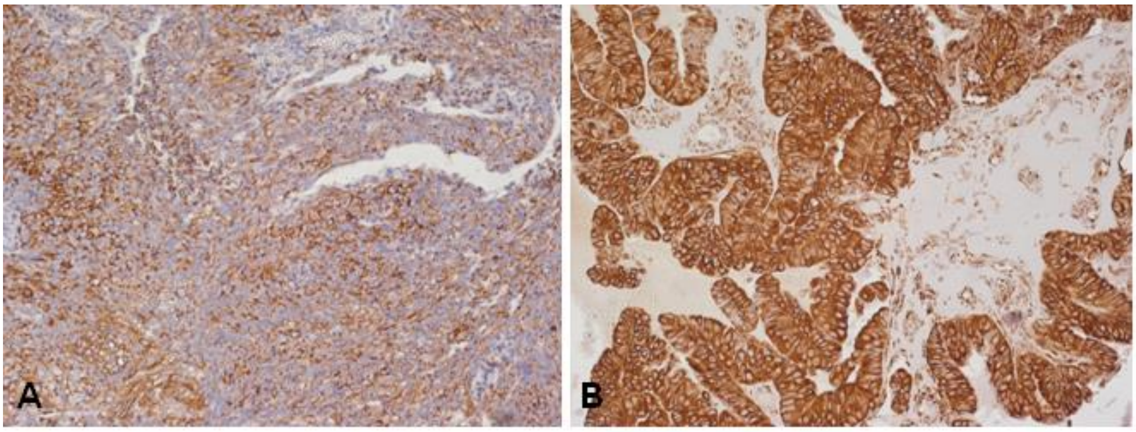

Supplemental Figure 4: B7-H4 expression in endometrial biopsies. Endometrial carcinoma biopsies showing A. moderate B7-H4 expression (20x enlarged) and B. strong expression level (20x enlarged) with in addition very clear cytoplasmic and circumferential B7-H4 presence
